# Sphingolipids in Lung Pathology in the Coronavirus Disease Era: A Review of Sphingolipid Involvement in the Pathogenesis of Lung Damage

**DOI:** 10.3389/fphys.2021.760638

**Published:** 2021-10-08

**Authors:** Saad A. Khan, Kayla F. Goliwas, Jessy S. Deshane

**Affiliations:** Division of Pulmonary, Allergy, and Critical Care Medicine, Department of Medicine, University of Alabama at Birmingham, Birmingham, AL, United States

**Keywords:** sphingolipid, COVID, cytokine storm, S1P pathway, lung disease

## Abstract

Sphingolipids are bioactive lipids involved in the regulation of cell survival, proliferation, and the inflammatory response. The SphK/S1P/S1PR pathway (S1P pathway) is a driver of many anti-apoptotic and proliferative processes. Pro-survival sphingolipid sphingosine-1-phosphate (S1P) initiates its signaling cascade by interacting with various sphingosine-1-phosphate receptors (S1PR) through which it is able to exert its pro-survival or inflammatory effects. Whereas sphingolipids, including ceramides and sphingosines are pro-apoptotic. The pro-apoptotic lipid, ceramide, can be produced *de novo* by ceramide synthases and converted to sphingosine by way of ceramidases. The balance of these antagonistic lipids and how this balance manifests is the essence of the sphingolipid rheostat. Recent studies on SARS-CoV-2 have implicated the S1P pathway in the pathogenesis of novel coronavirus disease COVID-19-related lung damage. Accumulating evidence indicates that an aberrant inflammatory process, known as “cytokine storm” causes lung injury in COVID-19, and studies have shown that the S1P pathway is involved in signaling this hyperinflammatory response. Beyond the influence of this pathway on cytokine storm, over the last decade the S1P pathway has been investigated for its role in a wide array of lung pathologies, including pulmonary fibrosis, pulmonary arterial hypertension (PAH), and lung cancer. Various studies have used S1P pathway modulators in models of lung disease; many of these efforts have yielded results that point to the potential efficacy of targeting this pathway for future treatment options. Additionally, they have emphasized S1P pathway’s significant role in inflammation, fibrosis, and a number of other endothelial and epithelial changes that contribute to lung damage. This review summarizes the S1P pathway’s involvement in COVID-19 and chronic lung diseases and discusses the potential for targeting S1P pathway as a therapeutic option for these diseases.

## Introduction

Sphingolipids are a class of lipids with a sphingoid backbone that are involved in cell signaling for a variety of catabolic and anabolic pathways. Sphingolipids often act as structural components of membranes, where they function as regulators of communication and signaling. Many bioactive mediators are formed in sphingolipid metabolism that act as secondary messengers and regulate cell functions such as migration, proliferation, apoptosis, and other pro- and anti-inflammatory processes (Chen et al., 2019; Suryadevara et al., 2020).

The disruption of sphingolipid metabolism has been shown to be implicated in the pathogenesis of several chronic lung diseases, including pulmonary fibrosis, pulmonary arterial hypertension (PAH), lung cancer, and others (Chen et al., 2014; Balaji Ragunathrao et al., 2019; Gachechiladze et al., 2019; Yan et al., 2019; Huang et al., 2020; McGowan et al., 2020). The lung actively metabolizes lipids constantly for normal physiologic function. Dysregulation of this metabolism alters the lung tissue and initiates the inflammatory process that drives the pathogenesis of chronic lung diseases (Agudelo et al., 2020).

The outbreak of the SARS-CoV-2 mediated novel coronavirus disease (COVID-19) has emerged as threat to health globally. Clinical outcomes for patients who contract the disease differ depending upon age and comorbidities (Ejaz et al., 2020). Evidence shows that COVID-19 has pathologic implications that coincide with a multitude of conditions, including pulmonary fibrosis, PAH, and lung cancer (Chen et al., 2014; Gachechiladze et al., 2019; Huang et al., 2020). Accumulating evidence suggests than an aberrant inflammatory process known as “cytokine storm” causes the lung injury in COVID-19. A sphingolipid signaling pathway known as the SphK1/S1P/S1PR (S1P Pathway) has been implicated in the onset of this hyperinflammatory response (McGowan et al., 2020).

The involvement of sphingolipid metabolic dysregulation in both COVID-19 and various chronic lung diseases suggests the possibility of synergistic damage to the lungs. Additionally, it suggests the potential to target sphingolipid metabolism as a therapeutic approach. The S1P pathway in particular is of great interest given its involvement in cell survival, proliferation, and inflammation (Hannun and Obeid, 2008). This review will discuss the involvement and therapeutic potential of sphingolipid metabolism in chronic lung diseases and COVID-19.

## Sphingolipid Pathway

**FIGURE 1:**
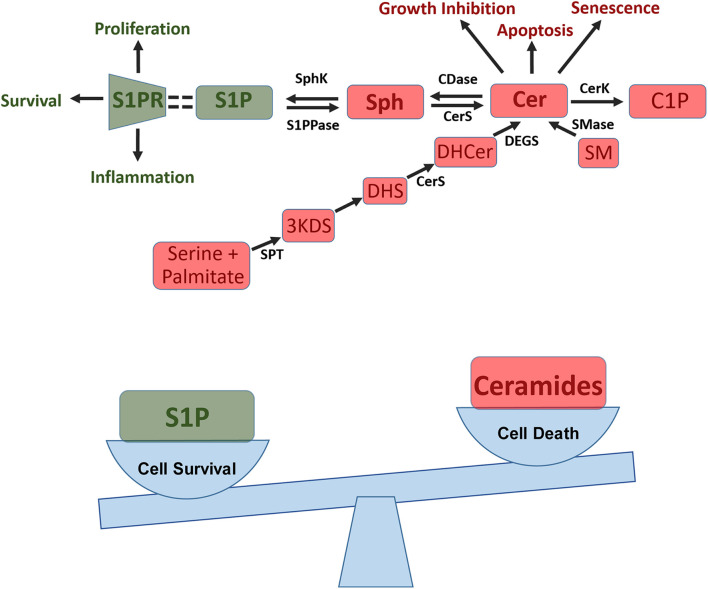
Sphingosine-1-phosphate (S1P) pathway and the sphingolipid rheostat. Top: S1P pathway summary showing how ceramide (Cer) is formed *de novo* from conversion of serine and palmitate into 3-keto-dihydrosphingosine (3KD) via serine palmitoyl transferases (SPT). 3KD is then reduced to dihydrosphingosine (DHS) and converted to dihydroceramide (DHCer) by ceramide synthase (CerS). Finally DHCer is converted to Cer by dihydrogceramide desaturase (DEGS). Cer can also be produced from sphingomyelin (SM) in the salvage pathway through sphingomyelinase (SMase). Cer can then be used to generate ceramide-1-phosphate (C1P) and can impact apoptosis, senescence, and growth inhibition. Additionally, Cer can be converted to sphingosine (Sph) via ceramidase (CDase), this process can then be reversed via CerS. Sph can then be converted into S1P via sphingosine kinases (SphK), a process which can be reversed via S1P phosphatase (S1PPase). S1P then binds to S1P receptors (S1PR) on cells to induce proliferation, cell survival, and inflammation. Bottom: Sphingolipid rheostat showing how the balance between pro-survival S1P and pro-apoptotic ceramide influences a cells ability to survive and proliferate.

The sphingolipid rheostat, summarized in Figure 1, is a concept that describes the balance between ceramide and sphingosine-1-phosphate (S1P), two sphingolipids that are involved in opposing signaling pathways. Ceramide is generally antagonistic to growth, involved in cellular apoptosis, senescence, and growth inhibition. Whereas, S1P is involved in cell survival, proliferation, and inflammation (Hannun and Obeid, 2008). S1P is produced from the phosphorylation of sphingosine with production mediated by the enzyme sphingosine kinase (SphK). In humans, there are two SphK isoforms, SphK1 and SphK2, with important differences. SphK1 is found in the cytosol, and can translocate to the plasma membrane upon stimulation (Wattenberg et al., 2006; ter Braak et al., 2009; Siow and Wattenberg, 2011; Blankenbach et al., 2020). SphK2 exists in and goes back and forth between the cytosol and nucleus depending on cellular conditions; there are reports of SphK2 in mitochondria and the endoplasmic reticulum as well (Siow and Wattenberg, 2011; Chan and Pitson, 2013; Blankenbach et al., 2020). While both enzymes act primarily on sphingosine and dihydrosphingosine, SphK1 functions with higher catalytic efficiency while SphK2 is capable of inefficiently phosphorylating additional substrates (Liu et al., 2000; Siow and Wattenberg, 2011). The enzyme sphingosine-1-phosphate phosphatase (S1PPase) converts S1P into sphingosine. Sphingosine-1-phosphate lyase (S1P lyase) converts S1P to ethanolamine phosphate and hexadecenal. Together these enzymes control levels of S1P (Kwong et al., 2015). S1P interacts with S1P receptors (S1PR), signaling inflammatory and growth responses. There are 5 S1PRs (S1PR1-5) with both unique and overlapping functions. Receptor expression varies based on cell type (Adada et al., 2013; Li et al., 2015; Li Q. et al., 2021). S1PR1-3 are the most commonly expressed subtypes and are present on endothelial cells (Adada et al., 2013; Li et al., 2015; Li Q. et al., 2021). S1PR1 is critical for the mobilization of lymphocytes from lymphoid tissue and is also involved in angiogenesis, proinflammatory signaling, and maintenance of the endothelial barrier among other things (Adada et al., 2013; Li et al., 2015; Li Q. et al., 2021). S1PR2 increases permeability of endothelial cell junctions, and is present in immune response, muscle function, and many organ systems (Adada et al., 2013; Li et al., 2015; Li Q. et al., 2021). S1PR3 is essential for the immune response and is present on dendritic cells; it can replace S1PR1 function in the setting of low S1PR1 expression (Adada et al., 2013; Li et al., 2015; Li Q. et al., 2021). S1PR4 is primarily involved in hematopoiesis (Adada et al., 2013). S1PR5 is primarily present on oligodendrocytes in the brain and may also contribute to the integrity of the blood brain barrier. Recently, the presence of S1PR5 in NK cells has become a point of discussion as well (Adada et al., 2013). Other sphingolipids within this pathway, such as ceramide-1-phosphate (C1P), are also bioactive. C1P, which is generated through phosphorylation of ceramide by ceramide kinase (CerK), is believed to play an important role in inflammation and cellular homeostasis (Hannun and Obeid, 2008; Presa et al., 2020; Prakash et al., 2021).

Ceramides can be converted to sphingosine by ceramidase enzymes, including human alkaline phytoceramidase (haPHC), human alkaline ceramidase 1 (haCER1), and human alkaline ceramidase 2 (haCER2) (Xu et al., 2006) haCER1 and haCER2 use ceramide as a substrate and regulate levels of sphingosine and S1P within cells (Xu et al., 2006; Hannun and Obeid, 2008). HaCER2 expression is known to promote serum-independent proliferation of cells via S1P (Xu et al., 2006; Hannun and Obeid, 2008). HaPHC, and to some extent haCER1 and haCER2, also use other substrates such as phytoceramide and dihydroceramide (Xu et al., 2006; Hannun and Obeid, 2008). Ceramide can also be produced from sphingomyelin, mediated by sphingomyelinase in the salvage pathway and can be generated *de novo* (Prakash et al., 2021). In the *de novo* process, serine and palmitate are condensed via the enzyme serine palmitoyl transferase (SPT) into 3-keto-dihydrosphingosine. This product is reduced into dihydrosphingosine, and then acylated by a ceramide synthase (CerS). After this acylation, ceramide can be formed by way of desaturase enzymes (Hannun and Obeid, 2008).

**FIGURE 2:**
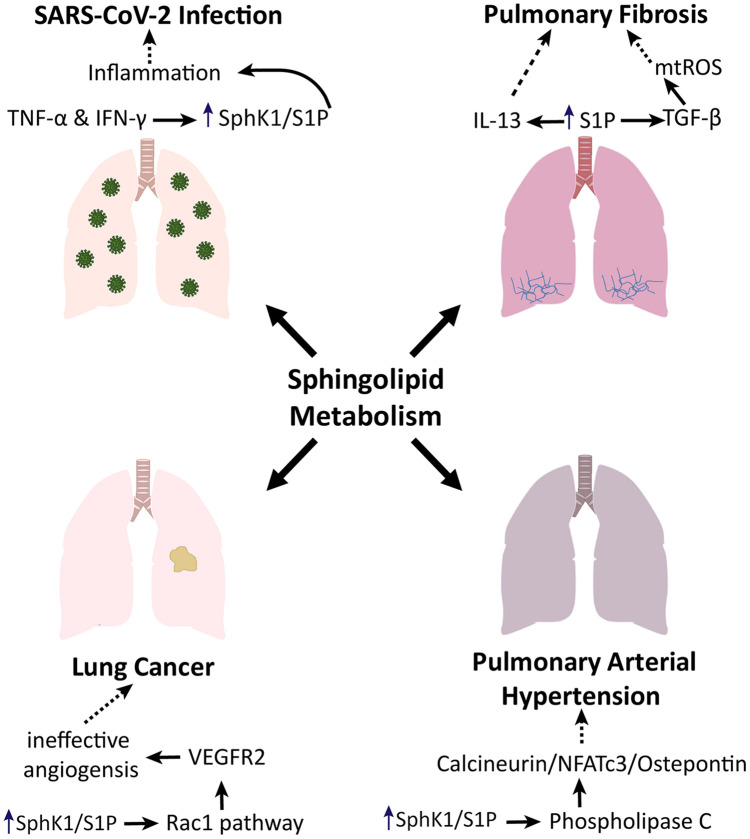
Sphingolipid metabolism in lung pathologies. Sphingolipid metabolism promotes pulmonary fibrosis via TGF- and IL-13 signaling. Sphingolipid metabolism induces phospholipase C to enhance the progression of pulmonary arterial hypertension. In the context of lung cancer, sphingolipid metabolism has been shown to impact angiogenesis via the Rac1 pathway. Additionally, sphingolipid metabolism is involved in the inflammatory phenotype associated with SARS-CoV-2 infection.

Due to the role of these sphingolipid pathways in cellular activity and inflammation, amongst other things, these pathways have become a point of interest in the treatment of several illnesses, including pulmonary fibrosis, PAH, lung cancer, and COVID-19, as shown in Figure 2.

## Sphingolipid Metabolism in Pulmonary Fibrosis

Recent rodent studies show that lung tissue with bleomycin-induced pulmonary fibrosis has increased SphK1 expression and S1P levels. The S1P pathway utilizes the S1PR1, S1PR2, and S1PR3 receptors on fibroblasts to influence this disease (Murakami et al., 2014; Park and Im, 2019; Huang et al., 2020). SphK1 deletion and S1PR2 deletion in lung fibroblasts have both been shown to be protective against bleomycin-induced pulmonary fibrosis in mice (Murakami et al., 2014; Park and Im, 2019; Huang et al., 2020). SIPR3 deletion in mouse models resulted in decreased fibrosis (Murakami et al., 2014). Huang et al. (2020) investigated the importance of this pathway in various cell types, and demonstrated that SphK1 is pro-inflammatory and pro-fibrotic in fibroblasts and alveolar epithelial cells, while it may be anti-inflammatory/anti-fibrotic in lung endothelial cells. In these models, the deletion of SphK1 also reduced markers of the Hippo pathway, a signaling pathway utilizing TGF-β and transcription factor YAP1 that is involved in cellular proliferation, differentiation, and lung fibrogenesis. They showed that YAP1 is upstream of mitochondrial reactive oxygen species (mtROS) generation. By limiting this pathway, there was a reduction in TGF-β mediated mtROS generation. MtROS production is necessary for TGF-β induced alpha-smooth muscle actin (α-SMA) and fibronectin (FN) production. S1P potentially modulates this entire pathway; use of an S1P antibody showed reduction in TGF-β mediated α-SMA and FN in lung fibroblasts. These findings demonstrate that TGF-β-induced YAP1 nuclear translocation in lung fibroblasts is dependent on intracellular S1P signaling (Huang et al., 2020).

Furthermore, JTE-013, an S1PR2 antagonist, reduced bleomycin-induced pulmonary fibrosis. S1PR2 may affect inflammatory response via modulation of TGF-β signaling, which induces epithelial-mesenchymal transition markers and extracellular matrix accumulation (Murakami et al., 2014). In another mouse model of lung fibrosis, investigation of the mechanism by which S1P induces fibrosis implicated the IL-13 pro-fibrotic cytokine. IL-13 acts through a downstream transcription factor known as STAT6. S1PR2 was found to contribute to increased IL-13 production and STAT6 phosphorylation in pulmonary macrophages (Zhao et al., 2018).

Fingolimod, an immunomodulator and a sphingosine analog, has been shown also to be pro-fibrotic (Keller et al., 2007; Potteck et al., 2010; Sobel et al., 2013; Gendron et al., 2017). Fingolimod may promote differentiation of lung fibroblasts (while also exerting an anti-apoptotic effect), cause increased extracellular matrix deposition, and influence growth factor expression which would suggest potential adverse effects of this pathway on fibrosis (Keller et al., 2007; Potteck et al., 2010; Sobel et al., 2013; Gendron et al., 2017). High doses of Fingolimod contribute to increased vascular leak that also worsens fibrosis and lung injury in animal models (Shea et al., 2010; Gendron et al., 2017). A study investigating these contradictory effects indicated that timing of administration in regards to disease course is paramount in eliciting a pro- or anti-fibrotic effect (Gendron et al., 2017). Results showed an anti-fibrotic effect when administered during the inflammatory stage, and a pro-fibrotic effect when administered during the remodeling stage (Gendron et al., 2017). The S1P pathway appears to be involved in the pathogenesis of pulmonary fibrosis through a myriad of different processes, indicating the potential efficacy of S1P pathway modulation in the treatment of pulmonary fibrosis.

## Sphingolipid Metabolism in Pulmonary Arterial Hypertension

Studies have examined the involvement of the SphK1 pathway in the pathology of PAH. S1P has been shown to utilize two specific receptors (S1PR2 and S1PR3) present on pulmonary arterial smooth muscle cells (PASMCs), with S1PR2 more strongly associated with the development of PAH (Yan et al., 2019). Flux through this pathway induced PASMC proliferation and ultimately the pulmonary vascular remodeling that is pathologic in PAH (Chen et al., 2014; Yan et al., 2019). Yan et al. (2019) demonstrated the mechanism of S1P-dependent PASMC proliferation, wherein S1P activates phospholipase C (PLC) in PASMCs, causing the release of calcium from the endoplasmic reticulum. S1P induced intracellular calcium release through PLC is also known to occur in adrenocortical cells and vascular smooth muscle cells (Yan et al., 2019). Inhibition of PLC in endothelial cells implicated S1PR1 specifically and confirmed that PLC (alongside inositol triphosphate) triggers intracellular calcium release (Li et al., 2015; Yan et al., 2019). Overall, studies indicate that S1P induction of calcium release is PLC dependent (Yoon et al., 2008; Li et al., 2015; Yan et al., 2019). This increased intracellular calcium activates the calcineurin/NFATc3/Osteopontin pathway. Osteopontin is a cytokine that has been shown to be elevated in heart failure models and has been proposed to be involved in the development of idiopathic and hypoxic PAH (Yan et al., 2019).

Lung SphK1 expression is greater in mice with PAH and knockout of SphK1 in mouse models is protective against hypoxia-induced PAH. Chen et al. (2014) used SphKI2, an inhibitor of SphK1 and SphK2, in mouse models of PAH, and noted reduced right ventricular pressure (RVP), right ventricular hypertrophy (RVH), and pulmonary vascular remodeling compared to controls. These findings suggest S1P involvement in the development of PAH. MacRitchie et al. (2016) implemented inhibitors of this pathway to examine the effect on pulmonary remodeling in mouse models of hypoxia-induced PAH. Additionally, use of PF-543 (a selective SphK1 inhibitor) led to significantly reduced RVH, but failed to reduce vascular remodeling. The lack of change in pulmonary vascular remodeling after selectively inhibiting SphK1 was suggested to be due to compensatory activity of SphK2 (MacRitchie et al., 2016). These studies collectively implicate S1P pathway in the pathogenesis of PAH.

## Sphingolipid Metabolism in Lung Cancer

Contribution of metabolism of sphingolipids in cancer has increasingly become recognized. Sphingolipids play important roles in the regulation of cancer cell signaling to control suppression of tumor or survival (Ponnusamy et al., 2010; Ogretmen, 2018). Ceramide has been shown to mediate cell death, whereas S1P induces tumor cell proliferation, resistance to chemotherapy, radiotherapy or immunotherapy and metastasis (Bektas et al., 2005; Pchejetski et al., 2005; Ponnusamy et al., 2010; Liu et al., 2011; Stefanovic et al., 2016; Ogretmen, 2018). Increased generation and accumulation of ceramides mediates cancer cell death via apoptosis, necroptosis or other mechanisms (Saddoughi and Ogretmen, 2013; Saddoughi et al., 2013; Ogretmen, 2018). Downstream mechanisms by which ceramide induces cell death are regulated by its subcellular localization, trafficking and lipid-protein binding between ceramide and target proteins in cancer cells (Chalfant et al., 2001; Kitatani et al., 2006; Mukhopadhyay et al., 2009; Ponnusamy et al., 2010; Ogretmen, 2018). The metabolic conversion of ceramide to S1P has been shown to increase cancer cell survival via S1PR-dependent or S1PR-independent oncogenic signaling (Hait et al., 2009; Alvarez et al., 2010; Strub et al., 2011; Xiong et al., 2013; Etemadi et al., 2015; Panneer Selvam et al., 2015; Parham et al., 2015; Ogretmen, 2018). Systemic S1P has been shown to mediate cell-cell communication between host cells and cancer cells to promote various aspects of metastasis of tumor, including S1P secretion from lymphoid endothelial cells and S1PR1-dependent or S1PR2-dependent signaling in cancer cells to induce migration and/or evade immune-cell-mediated cytotoxicity (Mao et al., 2010; Ponnusamy et al., 2012; van der Weyden et al., 2017; Ogretmen, 2018).

SphK1 and S1P have also been shown to have implications in lung cancer. SphK1 expression is significantly increased in non-small-cell lung cancer (NSCLC) tissue compared to normal tissue (Gachechiladze et al., 2019). SphK1 expression is higher at both the protein and the mRNA level; mRNA levels are nearly doubled in lung cancer tissue compared to normal tissue (Johnson et al., 2005). Increased SphK1 expression in NSCLC was associated with poorer survival in patients receiving platinum-based chemotherapy; there is a strong positive association between SphK1 levels and more advanced disease (Gachechiladze et al., 2019). These findings propose SphK1 as a potential prognostic indicator both in lung cancer and in the effectiveness of adjuvant platinum-based chemotherapy (Gachechiladze et al., 2019).

S1P pathway is involved in various malignancies; this pathway’s contribution to other malignancies provides potential insight on its role in lung cancer. Modulation of S1P levels has been examined to determine its effects on prostate cancer. Both systemic and tumor S1P have been shown to contribute to regulation of local tumor growth (Ponnusamy et al., 2012). However, only modulation of systemic SphK1 (not tumor SphK1), prevented S1P elevation and inhibited induced prostate cancer growth and lung metastasis (Ponnusamy et al., 2012). Additionally, a genetic loss of SphK1 led to an activation of a metastasis suppressor known as Breast Carcinoma Metastasis Suppressor 1 (Brms1) via S1PR2 in cancer cells (Ponnusamy et al., 2012). Sphingomab (an anti-S1P monoclonal antibody) was used to inhibit systemic S1P signaling, which suppressed lung metastasis. Knockout of Brms1 eliminated the metastasis suppressing effect of Sphingomab (Ponnusamy et al., 2012). S1PR1 has been implicated in lung cancer as well (Balaji Ragunathrao et al., 2019). S1P and S1PR1 are highly expressed in various lung tumors and can induce the Rac1 pathway to promote angiogenic signaling, specifically endothelial cell (EC) migration (Balaji Ragunathrao et al., 2019). Use of small interfering RNA (siRNA) to interrupt S1PR1 in ECs resulted in significantly diminished VEGF-induced angiogenesis and cell migration (Balaji Ragunathrao et al., 2019). Comparison of tumor associated ECs with and without S1PR1 showed impaired angiogenic signaling in the S1PR1 null ECs (Balaji Ragunathrao et al., 2019). Overall, S1PR1 promotes tumor growth by increasing VEGFR2 expression on the surface of endothelial cells, and thus increasing VEGFR2-mediated angiogenic signaling (Balaji Ragunathrao et al., 2019). VEGFR2 is known to lead to angiogenesis via activation of Rac1, ERK, and AKT (Lanahan et al., 2010, 2013; Ren et al., 2010; Claesson-Welsh and Welsh, 2013; Kofler and Simons, 2015; Balaji Ragunathrao et al., 2019). In order to activate ERK and AKT, VEGFR2 must undergo tyrosine residue phosphorylation, which occurs downstream of S1PR1 activation (Lanahan et al., 2010, 2013; Ren et al., 2010; Balaji Ragunathrao et al., 2019). Two different tyrosine residues have both been implicated in angiogenesis; Y1175 and Y951 (Lanahan et al., 2010, 2013; Ren et al., 2010; Balaji Ragunathrao et al., 2019). S1PR1-mediated VEGFR2 phosphorylation at the Y951 residue may be responsible for EC migration (Balaji Ragunathrao et al., 2019). Interference with S1PR1 caused a decrease in Rac1 activity (Balaji Ragunathrao et al., 2019). S1PR1 signaling leads to phosphorylation of VEGFR2 at Y951, but leaves Y1175 unchanged (Balaji Ragunathrao et al., 2019). In ECs without S1PR1, phosphorylation failed to occur at Y951, but increased at Y1175 (Balaji Ragunathrao et al., 2019). These results demonstrate that S1PR1 activity causes downstream effects via phosphorylation of Y951 (Balaji Ragunathrao et al., 2019). Use of a mutant Y951F-VEGFR2 that could not be phosphorylated at the Y951 residue led to defective endothelial cell migration compared to cells with non-mutated Y951 (Balaji Ragunathrao et al., 2019). Using this same mutant receptor allowed for the activities of Y951 and Y1175 to be distinguishable (Balaji Ragunathrao et al., 2019). Therefore, the activity of VEGFR2 with and without S1PR1 was observable (Balaji Ragunathrao et al., 2019). Presence of S1PR1 leads to VEGFR2 phosphorylation at Y951, which retains VEGFR2 at the EC surface and promotes migration and tumor growth via sustained Rac1 activity (Balaji Ragunathrao et al., 2019). The absence of S1PR1 leads to Y1175 phosphorylation, a subsequent internalization of VEGFR2, ephemeral Rac1 activity, and strong ERK1 activity which results in ineffective tumor angiogenesis (Balaji Ragunathrao et al., 2019). This ultimately indicates that activation of endothelial S1PR1 can lead to VEGFR2-induced angiogenesis (Balaji Ragunathrao et al., 2019). While previous studies using VEGF-VEGFR2 based cancer therapies do not demonstrate reduction of cancer recurrence, targeting both VEGFR2 and S1PR1 have resulted in successful reduction in cancer and cancer recurrence (Balaji Ragunathrao et al., 2019).

## Sphingolipids in Coronavirus Disease

### Coronavirus Disease Pandemic

Since the first reported cases of SARS-CoV-2 in 2019 until July 2021, the World Health Organization (WHO) reports upwards of 191 million cases, and over 4 million deaths worldwide (World Health Organization, 2021). While vaccination efforts have been strong, there has been a simultaneous emergence of new variants of SARS-CoV-2 which vary in viral structure, transmissibility, and disease severity (World Health Organization, 2020; Boehm et al., 2021). The WHO officially recognizes 4 variants of concern, with many additional variants being reported worldwide (World Health Organization, 2020, 2021). These recognized variants of concern are the Alpha, Beta, Gamma, and Delta variants. The Delta variant in particular, which was first identified in India in 2020 and was the dominant strain involved in the surge in India in April 2021, has become a point of discussion (Cascella et al., 2021; Lopez Bernal et al., 2021). The first reports of the Delta variant in the US was March 2021, and as of July has been reported in 124 countries (Cascella et al., 2021; Lopez Bernal et al., 2021; WHO, 2021). They also report an expectation for new variants to continue to arise (World Health Organization, 2020). Research is still emerging in terms of the efficacy of existing vaccines on novel strains of COVID-19 (Center for Disease Control and Prevention, 2021). Regardless, COVID-19 appears to be part of the public health landscape for the foreseeable future. As such, its impact and its long-term sequelae in conjunction with other chronic illnesses will need to be investigated and managed. A starting point for this is investigating the pathophysiology of damage caused by the virus.

### Pathway of Infection

Coronavirus SARS-CoV-2 infection depends on the virus’s spike protein (S) binding to angiotensin-converting enzyme 2 (ACE2) (Hoffmann et al., 2020; Kalfaoglu et al., 2020; Hu et al., 2021). Infection is also dependent on S protein priming by transmembrane serine protease 2 (TMPRSS2) (Hoffmann et al., 2020; Hussman, 2020). Infected individuals generally experience flu-like symptoms initially, such as cough, fever, and fatigue. Patients who are affected more severely may develop acute respiratory distress syndrome (ARDS), a severe lung injury characterized by widespread alveolar damage, hypoxemia, and bilateral infiltrates on imaging (Batah and Fabro, 2021). Studies point to a persistent cytokine release process, known as the cytokine storm, to be the predominant mechanism underlying immune-related damage in COVID-19 (McGowan et al., 2020; Rothan and Byrareddy, 2020; Hu et al., 2021).

### Cytokine Storm and Leukocytic Dyscrasias

Cytokine storm is a potentially lethal immune response characterized by abundant cytokine production that is known to be modulated, at least in part, by S1PR1 (Teijaro et al., 2014; Hoffmann et al., 2020; Hu et al., 2021). ACE2 is highly expressed in lung epithelial cells, particularly type-II alveolar pneumocytes. Infection with SARS-CoV-2 causes elevated production of pro-inflammatory cytokines such as tumor necrosis factor α (TNF-α), interleukin 6 (IL-6), and interleukin 12 (IL-12) which potentiate the innate immune response (Hussman, 2020; Hu et al., 2021). SARS-CoV-2 infection also causes rapid activation of pathogenic T helper type 1 (Th1) lymphocytes that co-express interferon γ (IFN-γ) and granulocyte-macrophage colony stimulating factor (GM-CSF). These GM-CSF^+^IFN-γ^+^ Th1 cells induce CD14^+^CD16^+^ monocytes that highly express IL-6, and therefore cause further inflammation (Zhou et al., 2020; Hu et al., 2021). One model proposes that IFN-γ release activates alveolar macrophages, which in turn become infected with SARS-CoV-2, and create a signaling loop with T cells that amplifies and sustains the release of inflammatory mediators and cytokines (Grant et al., 2021). COVID-19 patients exhibit lymphopenia and neutrophilia. Increased T cell activation, proliferation and cytotoxicity are present in severe disease. T cells in severe COVID-19 infection produce greater levels of cytotoxic granzyme B and perforin than healthy or less severe patients (Kang et al., 2020; Kalfaoglu et al., 2021). Inflammatory cytokines such as TNF-α and IFN-γ are known to induce SphK1 activation and increase intracellular S1P generation, which in turn promotes cell survival and various pro-inflammatory mediators (Alvarez et al., 2010).

Several studies have discovered progressive lymphopenia and increasing neutrophil-to-lymphocyte ratio in severe disease (Liu et al., 2020a; Ma et al., 2020; Bobcakova et al., 2021). Some severe cases of COVID-19 have shown significant increases in leukocyte and neutrophil counts, but decreased total lymphocytes (Li C.H. et al., 2021). Hyperactivation of the immune response may lead to more severe disease, ultimately leading to T cell exhaustion and lymphopenia (Bobcakova et al., 2021; Grant et al., 2021). Lymphopenia and neutrophilia in COVID-19 infection are possibly related to the pathogenic Th1 cell induction of CD14^+^CD16^+^ monocytes. These monocytes are correlated with increased neutrophil proliferation and decreased Th2 count (Hoffmann et al., 2020; Bossardi Ramos and Adam, 2021).

Walsh et al. (2014) demonstrated the efficacy of modulating the S1P Pathway; by administering antibodies to TNF-α and IFN-γ in rodent models, cytokine storm was greatly inhibited. They propose that direct S1P1R signaling on T cells are a potential component in the modulation of cytokine storm within this disease process. It remains to be seen if targeting SphK1 will suppress cytokine storm in the setting of COVID-19 (Walsh et al., 2014). However, cytokine storm is known to involve the S1P pathway, which is implicated in the pathophysiology of several other lung diseases. This creates the possibility of compounding damage in patients with COVID-19 and a preexisting or concurrent lung disease; targeting of this pathway could therefore be valuable.

### Pulmonary Epithelial and Endothelial Damage

We previously discussed the propensity of SARS-CoV-2 to cause lung injury and ARDS in severe infection. The mechanism of lung damage in COVID-19 infection sheds light on the immunologic interplay between the virus and other lung disease.

A histopathological study of changes in COVID-19 related lung disease revealed several changes from normal lung tissue, including damage to endothelial cell and epithelial/alveolar cells (Pannone et al., 2021). Endothelial alterations in COVID-19 include a hypercoagulability due to increased factor VIII and von Willebrand factor (Escher et al., 2020; Pannone et al., 2021). Additionally, there are elevated levels of plasma angiotensin II in COVID-19 patients. Angiotensin II can lead to vascular permeability and further endothelial damage (Liu et al., 2020b; Pannone et al., 2021). The aggressive production of pro-inflammatory cytokines such as IL-6 and TNF-α disrupts normal anti-thrombotic and anti-inflammatory function of the endothelium, which can cause dysregulated complement activation (Pannone et al., 2021). Complement activation can mediate thrombotic microvascular injury in COVID-19 (Magro et al., 2020; Pannone et al., 2021). Histopathologic review also found epithelial damage in COVID-19 infection, particularly in alveolar cells. Epithelial damage can also contribute to the formation of microthrombi and vascular damage (Pannone et al., 2021). Alveolar epithelium is responsible for regulating coagulation and fibrinolysis; injury to alveolar epithelial cells can lead to release and activation of pro-fibrotic TGF-β (John et al., 2021).

Epithelial and endothelial integrity and function are important components of normal lung function. As noted, disruption and dysregulation of epithelial, endothelial, and other cells can manifest in the form of various lung pathologies, such as pulmonary fibrosis and PAH. COVID-19 causes abnormalities throughout the pulmonary system, which creates potential for infection to induce or exacerbate a lung injury such as pulmonary fibrosis, PAH, or lung cancer.

## Drugs and Treatments Targeting Lipid Metabolism

As mentioned, some studies have used S1P pathway modulators in pre-clinical experiments. Fingolimod is an immune modulator that acts on S1P receptors and has been used in the treatment of Multiple Sclerosis (MS) for a decade (Chew et al., 2016). Fingolimod is non-selective and is known to modulate S1P receptors 1–5, except for S1PR2 (Fronza et al., 2021). Anecdotal evidence suggests Fingolimod’s utility in SARS-CoV-2 infection, and its potential efficacy is being evaluated (Barzegar et al., 2020; Yousefi et al., 2021). Fingolimod modulates inflammatory response, and while the mechanisms are still under investigation, it has been suggested as potential treatment for a variety of pathologies including mesenteric ischemia, acute lung injury, traumatic brain injury-induced lung and heart injury, Alzheimer’s disease (AD), and Parkinson’s Disease (PD) (Natarajan et al., 2013; Jesko et al., 2019; Camp et al., 2020; Poti et al., 2020; Qian et al., 2020). In AD, Fingolimod treatment altered mRNA expression of enzymes and receptors involved in S1P and ceramide pathways in animal models (Jesko et al., 2019). Presence of the V717L transgene (as in some cases of familial or early onset AD) modifies the cortical and hippocampal response to Fingolimod treatment (Jesko et al., 2019). The administration of Fingolimod in mouse models of PD also indicated a neuroprotective effect as determined by a reduction in the loss of dopaminergic neurons and attenuation of motor deficits (Zhao et al., 2017). Recently, more drugs have been approved that target the S1P pathway. In 2019, the FDA approved Siponimod, an inhibitor selective for S1PR1 and S1PR5, which has shown effectiveness in MS treatment (U.S. Food and Drug Administration, 2019; Fronza et al., 2021). Ozanimod was approved for use in MS in 2020, and is selective for S1PR1 and S1PR5 as well (U.S. Food and Drug Administration, 2020; Fauzyah et al., 2021; Perez-Jeldres et al., 2021). In March 2021, Ponesimod, a highly selective inhibitor of S1PR1, was approved for patients with relapsing MS (Fronza et al., 2021; U.S. Food and Drug Administration, 2021). Studies have shown that Ponesimod may be useful in treatment of a variety of diseases such as Hepatitis B, graft versus host disease, and others (D’Ambrosio et al., 2016; Fauzyah et al., 2021). These four drugs modulate S1PRs and have been approved for clinical use.

While S1P pathway modulators have been used in MS for some time, recent studies have begun to demonstrate efficacy in different pathologies such as rheumatoid arthritis (RA), systemic lupus erythematosus (SLE), psoriasis, and inflammatory bowel disease (IBD) including ulcerative colitis (UC), and Crohn’s disease (CD) (Perez-Jeldres et al., 2021). A clinical trial with Ozanimod showed effectiveness in the treatment of moderate to severe UC (Sandborn et al., 2016; Perez-Jeldres et al., 2021). Other medications that are not currently approved have undergone trials in the treatment of IBD with varying degrees of success; these drugs include Mocravimod, Etrasimod, and Amiselimod (Perez-Jeldres et al., 2021). Ponesimod has shown potential in psoriasis treatment (Vaclavkova et al., 2014; Perez-Jeldres et al., 2021). Cenerimod, a selective S1PR1 modulator, demonstrated treatment potential and an acceptable safety profile in the treatment of SLE (Hermann et al., 2019; Perez-Jeldres et al., 2021). Amiselimod, another selective S1PR1 modulator, has also shown promise in SLE treatment (Tanaka et al., 2020; Perez-Jeldres et al., 2021). A great deal of S1P modulators are currently being evaluated in clinical trials for potential utility across a variety of diseases. These modulators include ceralifimod, sonepcizumab, LC51-0255, OPL-002, GSK20188682, and ASP4058 and are detailed in Table 1 (Moberly et al., 2012; Xu et al., 2014; Stepanovska and Huwiler, 2020; Perez-Jeldres et al., 2021).

**TABLE 1:** Listing of all clinical trials involving S1P modulators in any phase and with any status.

**Trial identifier**	**Drug name**	**Indication**	**Trial phase**	**Status**	**Trial site**	**Mechanism of action**
NCT01375179	KRP203	UC	Phase 2	Terminated	6 European Countries	Selective S1PR1 Agonist
NCT02047734	Ozanimod	Relapsing MS	Phase 3	Completed with results	Cross-continental; 21 countries	Selective S1PR1,5 agonist
NCT02294058	Ozanimod	MS	Phase 3	Completed with results	Cross-continental; 25 countries	Selective S1PR1,5 agonist
NCT01647516	Ozanimod	UC	Phase 2	Completed with results	Cross-continental; 15 countries	Selective S1PR1,5 agonist
NCT02531126	Ozanimod	UC	Phase 3	Active, Not recruiting	Cross-continental; 29 countries	Selective S1PR1,5 agonist
NCT01647516	Ozanimod	UC	Phase 2	Completed with results	Cross-continental; 15 countries	Selective S1PR1,5 agonist
NCT03915769	Ozanimod	UC	Phase 3	Recruiting	Japan	Selective S1PR1,5 agonist
NCT03467958	Ozanimod	Crohn’s disease	Phase 3	Recruiting	Cross-continental; 45 countries	Selective S1PR1,5 agonist
NCT03440385	Ozanimod	Crohn’s disease	Phase 3	Recruiting	Cross-continental; 28 countries	Selective S1PR1,5 agonist
NCT03464097	Ozanimod	Crohn’s disease	Phase 3	Recruiting	Cross-continental; 45 countries	Selective S1PR1,5 agonist
NCT02447302	Etrasimod	UC	Phase 2	Completed with results	Cross-continental; 21 countries	Selective S1PR1,4,5 agonist
NCT03950232	Etrasimod	UC	Phase 3	Recruiting	Cross-continental; 41 countries	Selective S1PR1,4,5 agonist
NCT04173273	Etrasimod	Crohn’s disease	Phase 2 + Phase 3	Recruiting	145	Selective S1PR1,4,5 agonist
NCT03155932	Etrasimod	PBC	Phase 2	Terminated	United States, Australia, New Zealand	Selective S1PR1,4,5 agonist
NCT03072953	Etrasimod	Pyoderma gangrenosum	Phase 2	Terminated with results	Australia, New Zealand	Selective S1PR1,4,5 agonist
NCT01987843	Amiselimod	Plaque psoriasis	Phase 2	Completed	8 European countries	Selective S1PR1 + S1PR5 functional antagonist
NCT04162769	Etrasimod	Atopic dermatitis	Phase 2	Active, not recruiting	United States	Selective S1PR1,4,5 agonist
NCT01294774	KRP203	SLE	Phase 2	Completed	Germany, Greece, Italy	Selective S1PR1,4,5 agonist + Partial S1PR3 agonist
NCT02472795	Cenerimod	SLE	Phase 1 + Phase 2	Completed with results	United States and 5 European countries	Selective S1PR1 agonist
NCT03742037	Cenerimod	SLE	Phase 2	Active, Not recruiting	Cross-continental; 21 countries	Selective S1PR1 agonist
NCT01081782	Ceralifimod	MS	Phase 2	Terminated with results	Cross-continental; 12 countries	S1PR1,5 agonist
NCT01226745	Ceralifimod	MS	Phase 2	Terminated with results	Cross-continental; 12 countries	S1PR1,5 agonist
NCT00767949	Sonepcizumab	Neovascular age related macular degeneration	Phase 1	Unknown	United States	Anti-S1P monoclonal antibody
NCT04096573	LC51-0255	UC	Phase 2	Not yet recruiting	Undecided	Selective S1PR1 Agonist
NCT04360343	LC51-0255	UC	Phase 1	Completed	Seoul National University College of Medicine and Hospital	Selective S1PR1 Agonist
NCT04451811	OPL-002	Healthy	Phase 1	Completed	Belfast, Northern Ireland, United Kingdom	Selective S1PR1 Agonist
NCT01387217	GSK2018682	MS	Phase 1	Completed	Australia	Selective S1PR1 Agonist
NCT01431937	GSK2018682	MS, Relapsing-remitting	Phase 1	Completed	Australia	Selective S1PR1 Agonist
NCT01998646	ASP4058	Healthy	Phase 1	Completed	Florida, United States Indiana, United States	Selective S1PR1 Agonist
NCT02447302	Etrasimod	UC	Phase 2	Completed with results	Cross-Continental; 22 countries	Selective S1PR1,4,5 agonist
NCT00903383	LX3305	RA	Phase 2	Completed with results	United States, Bulgaria, Czech, Hungary, Poland, Serbia	S1PR Lyase inhibitor
NCT04405102	Ozanimod	COVID-19	Phase 2	Recruiting	Canada	Selective S1PR1,5 agonist
NCT04280588	Fingolimod	COVID-19	Phase 2	Withdrawn	Fuzhou, China	S1P analog; non-selective S1PR agonist
NCT01093326	Ponesimod	MS	Phase 2	Active, Not recruiting	Cross-continental; 20 countries	Selective S1PR1 Agonist
NCT00852670	Ponesimod	Plaque psoriasis	Phase 2	Completed	Austria, France, Germany, Hungary, Serbia	Selective S1PR1 Agonist
NCT01208090	Ponesimod	Psoriasis	Phase 2	Completed	Cross-continental; 17 countries	Selective S1PR1 Agonist
NCT01006265	Ponesimod	MS	Phase 2	Completed with results	Cross-continental; 23 countries	Selective S1PR1 Agonist
**NCT02002390**	Fingolimod	Stroke, Vascular accident	Phase 2	Completed	Tianjin, China	S1P analog; non-selective S1PR1,3–5 agonist
NCT04517552	[11C]-CS1P1	Alzheimers disease tracer	Phase 1	Active, Not recruiting	Missouri, United States	S1PR1 PET Radio-pharmaceutical
NCT04629872	Fingolimod	Stroke, inflammation	Phase 2	Recruiting	The First Affiliated Hospital of Fujian Medical University	S1P analog; non-selective S1PR1,3–5 agonist
NCT04675762	Fingolimod	Stroke, inflammation	Phase 2	Recruiting	Zhejiang, China	S1P analog; non-selective S1PR1,3–5 agonist
NCT04280588	Fingolimod	COVID-19	Phase 2	Withdrawn	Fuzhou, China	S1P analog; non-selective S1PR1,3–5 agonist
NCT02490930	Fingolimod	Glioblastoma, Anaplastic astrocytoma	Early phase 1	Completed	Johns Hopkins University	S1P analog; non-selective S1PR1,3–5 agonist
NCT04718064	Fingolimod	Stroke	Not applicable	Not yet recruiting	Xuzhou Medical University	S1P analog; non-selective S1PR1,3–5 agonist
NCT03915769	Ozanimod	UC	Phase 3	Recruiting	Japan	Selective S1PR1,5 agonist
NCT01334255	Sonepcizumab	Pigment epithelial detachment	Phase 1	Terminated	United States	Anti-S1P monoclonal antibody
NCT01488513	ABC294640	Pancreatic cancer, Other solid tumors	Phase 1	Completed	South Carolina, United States	Selective SphK2 inhibitor
NCT02229981	ABC294640	DLBCL, Kaposi sarcoma	Phase 1 + Phase 2	Withdrawn	Louisiana, United States	Selective SphK2 inhibitor
NCT03338998	Siponimod	Hemorrhagic stroke, ICH	Phase 2	Completed with results	United States	Selective S1PR1,5 agonist
NCT02576717	Ozanimod	MS	Phase 3	Active, Not recruiting	Cross-continental; 27 countries +	Selective S1PR1,5 agonist
NCT02757326	ABC294640	Multiple myeloma	Phase 1 + Phase 2	Terminated	North Carolina, United States	Selective SphK2 inhibitor

*Includes trial identification number, the specific modulator being tested, and the disease setting of the trial. Trial site also included. Some studies with many sites required summaries for table formatting. Full site locations can be found within the study record detail. This is found by searching the trial identification number at clinicaltrials.gov. DLBCL, diffuse large B-cell lymphoma; ICH, intracranial hemorrhage; MS, multiple sclerosis; PBC, primary biliary cirrhosis; RA, rheumatoid arthritis; SLE, systemic lupus erythematosus; UC, ulcerative colitis.*

Targeting S1P pathway to treat lung pathology has gained increasing attention recently. Several studies examine the potential efficacy in S1P modulation to treat a variety of lung disease such as cancer metastasis, pulmonary fibrosis, and PAH (Ponnusamy et al., 2012; Chen et al., 2014; MacRitchie et al., 2016; Weichand et al., 2017; Li et al., 2018; Balaji Ragunathrao et al., 2019; Gachechiladze et al., 2019; Yan et al., 2019; Huang et al., 2020). It has also been proposed as a treatment approach for diseases associated with endothelial dysfunction and vascular permeability; processes that are present in lung diseases such as COVID-19, ARDS, PAH, pulmonary fibrosis, lung cancer, and many others (Poirier et al., 2020). The S1P pathway is central to the pathogenesis of many lung diseases including COVID-19. Promising research in regards to S1P pathway modulation as well as the pathway’s role within the pathogenesis of many lung diseases suggests that the S1P pathway may be a promising target for therapy in COVID-19 and many other diseases. Ozanimod is currently recruiting for a Phase 2 clinical trial examining its potential for use in COVID-19 treatment (United States National Library of Medicine, 2020). It is likely that many more drug trials will begin to test the potential of S1P pathway modulation in lung disease.

## Author Contributions

SK produced manuscript and organized existing research. JD provided idea and framework for manuscript, guidance throughout review process, guidance through writing process, and contributed to final manuscript. KG produced figures and contributed to final manuscript. All authors contributed to the article and approved the submitted version.

## Conflict of Interest

The authors declare that the research was conducted in the absence of any commercial or financial relationships that could be construed as a potential conflict of interest.

## Publisher’s Note

All claims expressed in this article are solely those of the authors and do not necessarily represent those of their affiliated organizations, or those of the publisher, the editors and the reviewers. Any product that may be evaluated in this article, or claim that may be made by its manufacturer, is not guaranteed or endorsed by the publisher.
